# Acute Increase in Blood αCGRP at Maximal Exercise and Its Association to Cardiorespiratory Fitness, Carbohydrate Oxidation and Work Performed: An Exploratory Study in Young Men

**DOI:** 10.3390/biology10080783

**Published:** 2021-08-17

**Authors:** Adolfo Aracil-Marco, José Manuel Sarabia, Diego Pastor, Silvia Guillén, Raúl López-Grueso, Juana Gallar, Manuel Moya-Ramón

**Affiliations:** 1 Instituto de Neurociencias, UMH-CSIC, Department of Sports Sciences, UMH, 03202 Elche, Spain; 2 Department of Sports Sciences, Centro de Investigación del Deporte, UMH, 03202 Elche, Spain; jsarabia@umh.es (J.M.S.); dpastor@umh.es (D.P.); 3 Centro de Investigación del Deporte, UMH, Hospital Universitario de Elda, 03600 Elda, Spain; silviaggmedicina@hotmail.com; 4 Centro de Investigación del Deporte, UMH, 03202 Elche, Spain; rlopezgrueso@gmail.com; 5 Instituto de Neurociencias, UMH-CSIC, Alicante Institute for Health and Biomedical Research (ISABIAL), 03550 San Juan de Alicante, Spain; juana.gallar@umh.es; 6 Department of Sports Sciences, Centro de Investigación del Deporte, UMH, Alicante Institute for Health and Biomedical Research (ISABIAL), 03202 Elche, Spain

**Keywords:** αCGRP, carbohydrate oxidation, power, cardiorespiratory fitness, VO_2max_

## Abstract

**Simple Summary:**

αCGRP is a neuropeptide that increases in blood during high-intensity exercise in humans. However, the physiological meaning of this molecular response is unknown. Previous experimental works in rodents have related this neuropeptide to several biological processes in the skeletal muscle tissue and cardiorespiratory physiology. Based on the data from these animal studies we hypothesized that in humans αCGRP release during exercise could be similarly associated to metabolic and cardiorespiratory responses. To test this hypothesis, we subjected a sample of physically active young men to an exercise test up to exhaustion while their oxygen uptake (VO_2max_), CO_2_ production (VCO_2_), carbohydrate oxidation and performed work were measured. Blood samples were taken before the exercise test, at maximal intensity and after the volunteers have recovered, and the blood concentration of αCGRP was measured. We found that 2/3 of the volunteers responded to maximal exercise with an increase of their blood αCGRP concentration (responders), while the resting 1/3 did not (non-responders). We also found that VO_2max_, VCO_2_, carbohydrate oxidation and performed work were higher in the responders when compared to the non-responders. Therefore, our observations support that αCGRP release during exercise may be associated to physiological responses related to physical performance.

**Abstract:**

This study aimed to explore if the acute variations in plasma concentration of α-calcitonin gene-related peptide (αCGRP) induced by a single maximal exercise bout may be associated to cardiorespiratory fitness and carbohydrate oxidation in humans. Twelve young adult Caucasian men (24.3 ± 0.9 years-old; 179.2 ± 1.9 cm of height; 23.9 ± 0.6 kg·m^−2^ body mass index) performed a graded exercise test. A venous catheter was placed before testing, and blood samples were taken at baseline, maximal effort and recovery. αCGRP was measured in plasma using a commercial double-sandwich enzyme-linked-immunoassay. A two-way repeated measurements ANOVA was used to compare the values obtained at baseline, maximal effort and recovery. In the whole sample, αCGRP increased at maximal effort and its concentration correlated directly, albeit non-significantly, with the muscle mass normalised VO_2_, VCO_2_, carbohydrate oxidation and relative power. Two thirds of the participants showed an increase in αCGRP concentration at maximal effort. Post hoc analysis showed that in these individuals, the muscle mass normalised VO_2_, VCO_2_, carbohydrate oxidation rate and relative power were higher than in the participants lacking this molecular response. Therefore, our data suggest that (a) a majority of young men respond to exercise with an increase in blood αCGRP concentration; and (b) individuals exhibiting this response also show a higher cardiorespiratory fitness, carbohydrate oxidation and work performed. These findings suggest that this neuropeptide could act as an exerkine with potential effects on physical performance.

## 1. Introduction

Alpha-calcitonin gene-related peptide (αCGRP) is a 37 amino-acid neuropeptide produced by the alternative splicing of the calcitonin gene in nervous tissues [1]. Although it is best known as a vasodilatador, to date, αCGRP is considered a pleiotropic molecule, with multiple effects in different tissues of the body [2]. Among its cardio-circulatory effects, αCGRP acts both as a direct [3] and an indirect [4] potent and long-lasting vasodilator, it exerts inotropic and chronotropic actions [5,6], promotes cardiac hypertrophy in response to exercise [7] and stimulates angiogenesis in the skeletal muscle [8]. All these factors may contribute to increase the oxygen delivery to the contracting skeletal muscle. Moreover, αCGRP exerts potent metabolic effects on the skeletal muscle fibres. For example, in isolated strips of rat skeletal muscle in vitro, Leighton and Cooper [9] demonstrated that this neuropeptide inhibited insulin-induced glycogenesis without affecting glycolysis or the transmembrane transport of a glucose analogue [9,10]. These effects were reversed with the use of the αCGRP antagonist ^8–37^hCGRP [11]. In addition, αCGRP increased glycolysis specifically in muscles predominantly constituted by type IIa skeletal muscle fibres [12]. In vivo, the administration of αCGRP to conscious rats antagonised multiple metabolic effects of insulin [13]. For example, it reverted the insulin-induced inhibition of hepatic gluconeogenesis and the stimulation of skeletal muscle glycogenesis. Glycogenesis inhibition in skeletal muscle by αCGRP, in turn, increased the muscle glucose-6-phosphate concentration [13]. More recently, it has been demonstrated that αCGRP decreases the content of triglycerides and increases the content of free fatty acids in rat skeletal muscle [14] with high pharmacological potency. This lipolytic effect is accompanied by the stimulation of skeletal muscle β-oxidation [14]. Therefore, αCGRP seems to increase the bioavailability of energy sources in skeletal muscle fibres. Additionally, at the motor end plate, αCGRP is co-released with acetylcholine upon α-motoneuron activation [15]. Presynaptically, αCGRP increases the quantal release of acetylcholine from motoneurons [16], and additionally, it reduces the acetylcholinesterase activity in fast-twitch muscles, [17], thus increasing the excitatory effect of acetylcholine on the skeletal muscle fibre. Postsynaptically, αCGRP stimulates the sarcolemma Na^+^/K^+^-ATPase pump [18], thus increasing the restoration of the membrane potential after depolarisation. Therefore, αCGRP increases the excitability of skeletal muscle fibres.

In resting humans, the blood concentration of αCGRP shows a large inter-individual variability (14.0–132.5 pg·mL^−1^) [19], but it seems to be very constant intra-individually [20]. Although baseline blood αCGRP seems to be unaffected by training [21], several independent works found that this neuropeptide transiently increases in the blood in response to exercise [21,22,23,24,25,26,27,28]. However, fifteen years after the description of this phenomenon [21], the physiological significance of αCGRP release during exercise is still unknown.

Therefore, given the wide range of cardiorespiratory and metabolic effects of αCGRP described in animal models, we hypothesised that exercise-induced αCGRP release would be associated to cardiorespiratory fitness and carbohydrate oxidation in humans, a topic that, to the best of our knowledge, has not yet been addressed. This exploratory study aimed to test this hypothesis by measuring blood αCGRP concentration, cardiorespiratory fitness, carbohydrate oxidation and work performed during a graded exercise test in young, active men.

## 2. Materials and Methods

### 2.1. Study Design

A cross-sectional study was performed with 12 healthy Caucasian young adult men (see Table 1 for a description) that voluntarily participated in the study after signing an informed consent. Sample size was based on previous works that aimed to study αCGRP release during exercise in laboratory settings, which ranged from 6 to 12 participants [21,25,26,27].

### 2.2. Anthropometric Measurements

Before starting the exercise test, the anthropometric characteristics of the participants were measured according to standardised validated protocols for the Spanish population [29] by a Level 2 ISAK certified anthropometrist. Body mass was measured using a digital scale with an accuracy of 0.1 kg (Tanita, TBF 300 A, Kyoto, Japan), height with a wall mounted stadiometer with an accuracy of 0.1 cm (Seca 222, Amburg, Germany), breadth with a Holtain bicondylar calliper (Holtain Ltd., Pembs, UK), girth with a metallic non-extensible tape (Lufkin, TX, USA) and skinfolds with a Holtain Tanner/Whitehouse skinfold calliper (Holtain Ltd., Pembs, UK). Following the specific guidelines for this population [29], the percentage of body fat mass was calculated with the Durnin–Womersley equation [30] and the percentage of muscle mass was calculated with Lee’s equation [31]. From the latter, muscle mass (MM) in kg was estimated.

### 2.3. Leisure Time Physical Activity (LPTA)

LTPA was estimated with the Spanish self-administered short version of the International Physical Activity Questionnaire [32], and expressed as MET-min·week^−1^. Briefly, three kinds of physical activities are considered in the scoring system of the instrument (low, moderate and high intensity, such as, for example, walking, riding a bike or running, respectively). The weekly time that the individual spends performing each of these activities is also registered. By multiplying the reported time per an assigned number of metabolic equivalents (METs) to each intensity level, the MET-min·week^−1^ of physical activity of each intensity are obtained. The total MET-min·week^−1^ is the summation of all the categories. To obtain these data, we used the Excel template that can be downloaded from the instrument’s creators.

### 2.4. Graded Exercise Test

The exercise test was performed by each participant in a single session in a laboratory with controlled temperature (~23 °C), located 80 m above the sea level, between 17:00 and 19:00, to avoid the circadian baseline changes of αCGRP [20]. The subjects were instructed to maintain their regular diet and physical activity habits except for preventing them from performing intense physical activity during the previous 48 h and avoiding caffeine and alcohol consumption in the 2–3 h before testing.

Oxygen uptake (VO_2_) and CO_2_ production (VCO_2_) were measured during a graded exercise test up to exhaustion (see below). For testing, an electromagnetically braked cycle ergometer (Monark 839 Ergomedic, Monark, Vansbro, Sweden), a calibrated gas exchange analyser (K4b^2^, COSMED, Srl., Albano Laziale, Italy) and an automated blood pressure monitor (Tango+, SunTech Medical, Morrisville, NC, USA) were used. Throughout the test, a 12-lead electrocardiogram and pulse-oximetry were continuously monitored. Heart rate was obtained from the electrocardiogram recording. Calorimetry and cardiorespiratory responses were indirectly calculated using the gas analyser software. VO_2_, VCO_2_, energy expenditure (EE), carbohydrate oxidation (CHO) as well as the relative power performed by the individual were normalised to his muscle mass (indicated with the sub-index MM throughout the text).

The test was specifically customised for this study, according to the recommendations of Pettitt et al. [33]. Briefly, the test started with a 10-minute rest on the ergometer, followed by a 10 W warm-up for 2 min. Immediately after this, resistance increased up to 50 W for 2 min, and in steps of 25 W every 2 min from then on, until exhaustion. Pedalling cadence was maintained at around 60 revolutions·min^−1^ using a metronome. Test was finished if any of these two conditions of exhaustion were reached: (a) the desire of the subject to stop (volitional exhaustion); or (b) the inability of the subject to maintain the target pedalling cadence. The criterion to consider the test as maximal was to reach a respiratory exchange ratio (RER) > 1.1 [34]. Testing ended with a 3-minute cool down phase, during which the subject pedalled at 10 W. Recovery was considered finished when the subject’s heart rate reached 50% of his heart rate reserve [35]. The time spent between the end of the test and the recovery was recorded. Blood pressure was measured during the second minute of every step. The first and second ventilatory thresholds (VT1 and VT2, respectively) were determined with the ventilatory equivalents method [36].

### 2.5. αCGRP Quantification

An intravenous catheter was placed in the right antecubital vein before starting the exercise test. The catheter was filled with sterile physiological saline. Blood samples were withdrawn at baseline (BAS), immediately after the exercise test ended (MAX) and at recovery (REC). On each occasion, the first 2 mL of blood were discarded, and the following 5 mL were collected in EDTA-containing tubes (Deltalab, Barcelona, Spain), gently mixed, aliquoted in 2-millilitre polypropylene tubes and centrifuged at 3000 rpm for 10 min. Supernatant plasma was recovered and stored at −80 °C until assayed. For assaying, plasma samples of each subject were slowly thawed at room temperature and homogenised by gentle inversion. According to the enzyme-immuno-assay manufacturer, αCGRP was extracted using Oasis HLB cartridges C18 (SPI-BIO, Montigny-le-Bretonneux, France), recovered in methanol:water (9:1, v:v) and vacuum centrifuged at 35 °C, until dried. Pellets were afterwards re-suspended in the enzyme-immuno-assay buffer, and subsequently assayed for human αCGRP concentration using a commercial double-sandwich enzyme-immuno-assay kit (SPI-BIO #A05481). The sample of each subject and the CGRP concentration standards were assayed in duplicate, and the mean value was used for statistics. Intra and inter-assay variations were within the limits indicated by the manufacturer. Assays were masked, i.e., the involved researchers did not know any data of the subjects.

Plasma volume variations were not expected due to the short duration of the exercise test and the controlled environment conditions. However, to exclude this possibility, some of the initial plasma samples were assayed in parallel for albumin concentration (Pierce BCA protein assay kit, Thermo Scientific, #23227, Waltham, MA, USA) at the three measurement points. No differences were noted in albumin concentration between them.

### 2.6. Statistical Analyses

Data were analysed, and graphs were drawn using Sigmaplot 11 (Systat Software GmbH, Erkrath, Germany) or Microsoft Excel™. Unless otherwise indicated, data are expressed as mean ± standard error of the mean. Anthropometric and LTPA data were compared with the Student’s *t*-test. A two-way repeated measurements ANOVA (2-way RM ANOVA) was used for between and within group comparison at the different measurement points taken throughout the test. The corresponding non-parametric analyses were applied when appropriate and are indicated in the text. Differences were considered significant for *p* < 0.05. To calculate the Effect Size (ES), Cohen’s d was used and was interpreted as follows: 0.20–0.50 (small), 0.50–0.80 (medium), >0.80 (large) [37]. ES is presented throughout the text with the corresponding 95% interval of confidence (IC95%).

### 2.7. Ethics

All the experimental procedures followed a protocol approved by the university Ethics Committee (Comité de Ética en la Investigación Experimental de la Universidad Miguel Hernández de Elche; code: DPS-MMR-001-10) and attained to the tenets of the Declaration of Helsinki, as well as to the current national and international regulations for research involving human beings.

## 3. Results

### 3.1. Descriptive Results of the Whole Sample

Overall, the increase in αCGRP concentration at maximal effort showed a moderate ES (44.0 ± 34.7, 60.0 ± 33.2 and 52.5 ± 44.4 pg·mL^−1^, BAS, MAX and REC, respectively; *p* = 0.138, 2-way RM ANOVA on Ranks; d = 0.59 (−0.12, 1.25)). Although not significantly, the αCGRP concentration at maximal effort tended to correlate directly with VO_2MM_, VCO_2MM_, CHO_MM_ and relative power (see Figure S1).

### 3.2. Post Hoc Comparison between ‘Responders’ and ‘Non-Responders’

**Figure 1:**
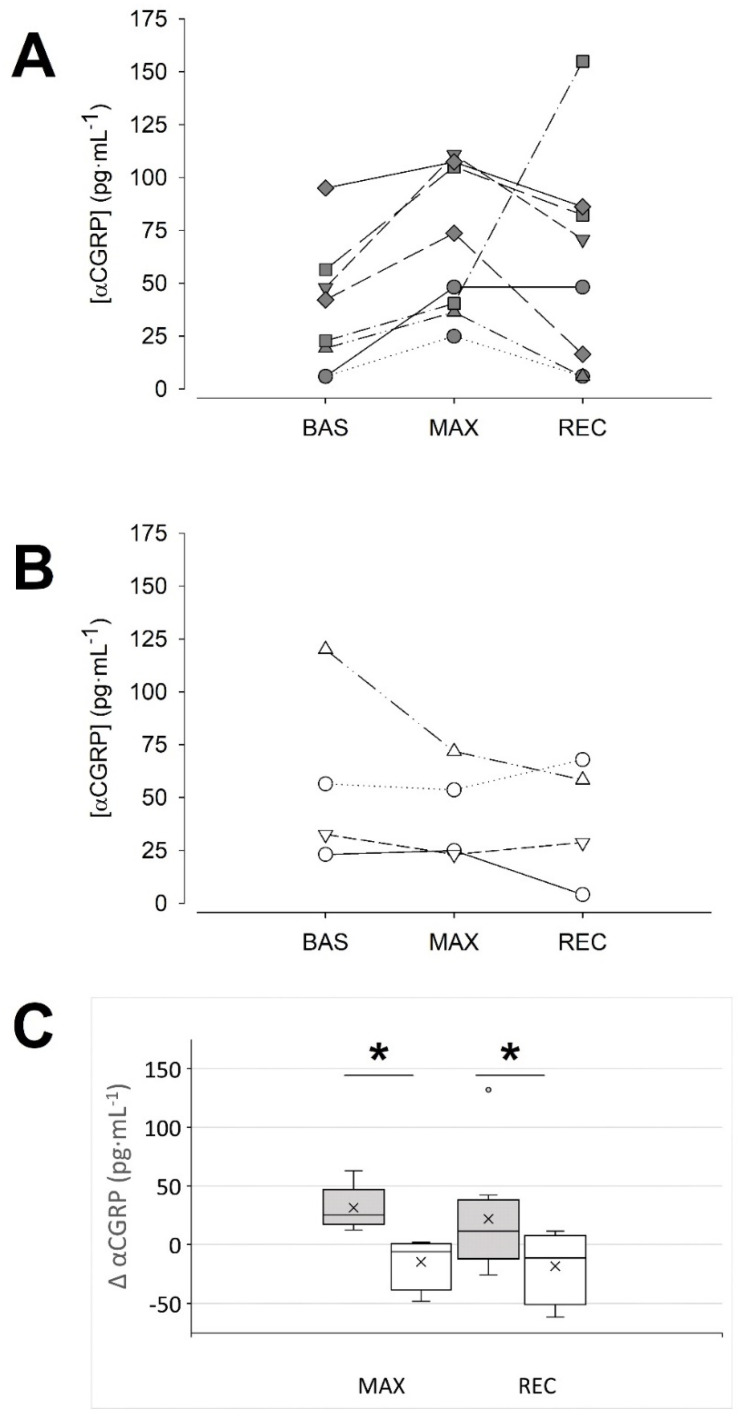
Two patterns of αCGRP response to maximal exercise could be identified. (**A**): data of the individuals that responded with an increase in their αCGRP concentration (‘responders’) at the maximal intensity. (**B**): data of the individuals in which blood αCGRP concentration was unaffected or tended to decrease at maximal intensity (‘non-responders’). (**C**): Absolute change of αCGRP concentration at the indicated condition vs. the BAS condition: dark grey boxes, ‘responders’, *n* = 8; white boxes, ‘non-responders’, *n* = 4; the cross represents the mean. * *p* < 0.05, two-way RM ANOVA. BAS: baseline; MAX: maximal effort; REC: recovery.

Unexpectedly, a detailed visual inspection of the data showed two response patterns. As shown in Figure 1, ~66% of the sample responded with an increase in αCGRP at MAX in comparison to BAS (36.9 ± 30.1, 68.3 ± 35.4 and 58.8 ± 50.9 pg·mL^−1^, BAS, MAX and REC, respectively; *p* = 0.01 MAX vs. BAS, Tukey test; d = 0.96 (0.04, 1.77)). Therefore, we named them the ‘responders’ group. In contrast, the ‘non-responders’ group (~33% of the sample, Figure 1B,C) did not show this response (58.0 ± 43.7, 43.4 ± 23.6 and 39.8 ± 29.0 pg·mL^−1^, BAS, MAX and REC, respectively; *p* = 0.350, 2-way RM ANOVA; MAX vs. BAS d = 0.42 (−0.81, 1.53)). No differences were found among both groups at baseline, neither in the αCGRP concentration (*p* = 0.345, Student’s *t*-test), nor in other variables (Table 1 and Table 2).

For further analyses, the data were grouped according to the αCGRP response at MAX. As shown in Table 2, the post hoc Tukey test showed that the responder group developed more work at the three measurement time points, and the percentage of energy coming from carbohydrate oxidation was higher in the responder group at VT1 (*p* < 0.01). Similarly, the CO_2_ production normalised to the individual’s weight was higher in the responder group at VT1 and MAX (*p* < 0.05). No other differences were noticed.

**Figure 2:**
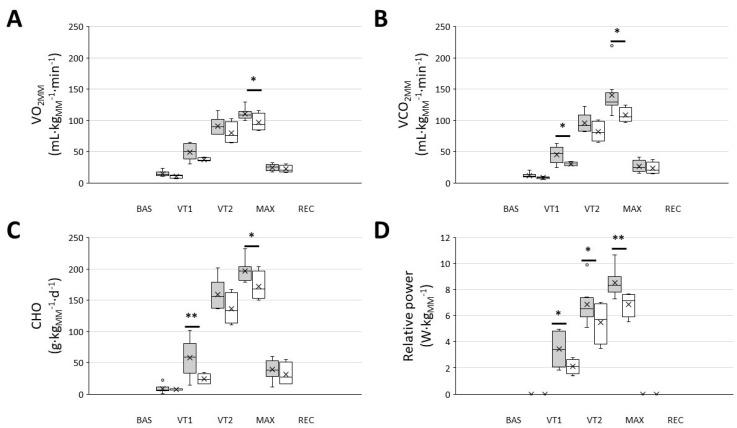
Men that were included in the ‘responders’ group showed a higher muscle mass normalised VO_2_, CO_2_, carbohydrate oxidation and relative power throughout the graded exercise ergospirometric test. (**A**): VO_2_ at different intensities; (**B**): VCO_2_ at different intensities; (**C**): carbohydrate oxidation (CHO) at different intensities; (**D**): work performed at different intensities. Dark-grey boxes, ‘responders’, *n* = 8; white boxes: ‘non-responders’, *n* = 4. The cross represents the mean. BAS: baseline; MAX: maximal effort; REC: recovery; VT1 and VT2: ventilatory thresholds 1 and 2, respectively; * *p* < 0.05, ** *p* < 0.01, 2-way RM ANOVA.

When the metabolic and performance variables were normalised to the active mass during exercise, i.e., the muscle mass, the responder group exhibited a significantly higher VO_2MM_, VCO_2MM_, energy from carbohydrate oxidation and relative power throughout the whole exercise test (Figure 2).

## 4. Discussion

This exploratory study aimed to test the hypothesis that αCGRP release during exercise may be associated to cardiorespiratory fitness and carbohydrate oxidation in humans. In agreement with previous works [21,23,25,26,27], in the present study, it has been observed that αCGRP increases during maximal exercise in healthy humans. The data also show a high individual variability in αCGRP blood concentration at the three measurement points taken. In addition, after a detailed data inspection, it was observed that this response was exhibited by approximately two thirds of the sample.

Due to the observational design of the present study, the mechanistic explanations of this response can be only hypothesised at present. Exercise intensity seems to be a determinant for the αCGRP release, since it is not evoked during sustained low-intensity exercise [21,25,28]. In a previous work, it has been observed that after maximal exercise, αCGRP release follows a close linear correlation with lactate concentration [25]. In contrast, Brooks et al. [38] could not find changes in blood αCGRP concentration in individuals performing 10 bouts of all-out 6 s sprint intervals with 30 s of recovery periods, despite a lactate concentration elevation, thus suggesting that the exercise-induced release of αCGRP may also be sensitive to other characteristics of the performed effort, such as its duration or its intervallic or continuous pattern, that can be, in turn, related to the bioenergetic system in use. In this regard, in a work aimed to relate αCGRP with exercise-induced migraine, Tarperi et al. have recently described that αCGRP increases 1.5-fold after a half-marathon performed at 75–85% VO_2max_ [23].

αCGRP has been also involved in the regulation of blood pressure [19]. Due to its potent vasodilator activity, this neuropeptide has been suggested to be a part of a negative loop counteracting blood pressure increases [39]. In addition to their release from α-motoneurons, αCGRP is synthetised by peripheral sensory neurons that are widely distributed along the blood vessels and express TRPV1 [2], a polymodal membrane receptor activated by mechanical forces when the temperature increases and by the acidification of the internal milieu [40]. Upon TRPV1 activation, these neurons release the peptide [2]. Therefore, during exercise, the increased blood pressure, core temperature and local acidosis may overlap as redundant physiological stimuli for the αCGRP release from sensory neurons. Interestingly, although the differences were not statistically significant, according to the data (Table 2), heart rate, stroke volume, cardiac output and systolic blood pressure tended to be higher in the responder group throughout the exercise test.

There is also a subset of young men that do not respond to maximal exercise with increases in αCGRP blood concentration. Looking in detail in previous published works, some similar data may be found. For example, Lind et al. [27] observed that in 5 out of 30 participants (i.e., 16%)—that included middle-aged healthy control subjects as well as hypertensive and diabetic patients—the αCGRP concentration was below the detection level of the radioimmunoassay that they used. These individuals were found in the three groups. Although the authors considered this finding as an absence of response, conceivably, a technical limitation could not be ruled out. Similarly, although it was not explicitly noticed by the authors, looking in depth at the data presented by Tarperi et al. [23], it can be observed that in roughly 20% of their sample, αCGRP did not increase after performing a half-marathon. However, the ecological setting of their work—the participants could drink ad libitum during the race, for example—makes the interpretation of this observation difficult. As a strength of the present work, data were obtained in a highly controlled setting, and despite the limited sample size, they showed that one in three of the participants did not respond with αCGRP blood increases to maximal exercise. Therefore, despite the differences among all of these works (study design, participants, methods, etc.), taking all of them together, the existence of a subset of individuals that do not respond to maximal exercise with αCGRP blood increases is plausible and deserves further studies.

The need to investigate the physiological significance of αCGRP blood increases in response to exercise was claimed since its first description in humans [21]. However, to the best of our knowledge there are no previous works specifically investigating it. Given the pleiotropic actions of this neuropeptide on cardiorespiratory physiology and muscle metabolism, the present work aimed to look for the putative association of αCGRP release during a single bout of exercise performed at maximal intensity with cardiorespiratory fitness and carbohydrate oxidation. Segregating the participants according to their αCGRP response is a novel approach to data analysis and allowed the observation that the release of this neuropeptide is associated to higher VO_2_, carbohydrate oxidation rate and relative power, normalised to the subject’s muscle mass, when exercising at maximal intensity.

Despite this, the present study has several limitations that deserve further consideration. First, given that this was an exploratory study, only a small sample was included, and some other potentially relevant variables, such as blood lactate, were not measured. In addition, similarly to the previous literature, in this work individuals were only measured once, and, therefore, it would be interesting in the future to study if the individual’s response is maintained over time. Experimental studies are also needed to clearly identify the causative factor of the association. Second, only young and healthy men were included and, therefore, the results might not be representative for the whole population (older population, sedentary subjects, women, etc.). Third, αCGRP was tested after a specific exercise test and the effect of other types of exercise or training programs could be different. Nonetheless, the study was designed with the very specific objective to respond to the initial hypothesis.

## 5. Conclusions

Among young men, a majority of individuals respond to maximal exercise with plasma αCGRP elevations. Individuals with this molecular response exhibit higher cardiorespiratory fitness, carbohydrate oxidation and work performed than individuals in which the response is absent. These findings suggest that αCGRP could be an exerkine with potential effects on physical performance.

## Figures and Tables

**Table 1:** Characteristics of the volunteers.

	Total (*n* = 12)	‘Responders’ (*n* = 8)	‘Non-Responders’ (*n* = 4)	*p*
**Age (years)**	24.3 ± 0.9	23.9 ± 1.2	25.0 ± 1.6	0.608 ^a^
**Anthropometry**				
BM (Kg)	76.7 ± 2.1	75.6 ± 3.2	77.2 ± 1.8	0.897
Height (cm)	179.2 ± 1.9	180.3 ± 2.0	174.4 ± 3.9	0.080
BMI (Kg·m^−2^)	23.9 ± 0.6	23.1 ± 0.6	25.4 ± 1.0	0.154
FM (%)	14.1 ± 0.6	14.0 ± 1.6	14.2 ± 1.2	0.866
MM (%)	44.9 ± 0.8	44.4 ± 1.2	45.9 ± 0.9	0.461 ^a^
**LTPA (MET-min·week^−1^)**				
Low intensity	1178.4 ± 394.9	1049.8 ± 404.9	1435.5 ± 957.7	0.667
Moderate intensity	805.7 ± 302.5	552.7 ± 347.2	1311.7 ± 562.0	0.368 ^a^
High intensity	1381.8 ± 214.8	1392.1 ± 307.6	1361.2 ± 256.8	0.950
Total	3365.9 ± 613.3	2994.6 ± 843.3	4108.5 ± 736.9	0.418

BM: body mass; BMI: body mass index; FM: fat mass; MM: muscle mass; LTPA: leisure time physical activity; MET: metabolic equivalent. Comparisons were made with the Student’s *t*-test, except when indicated with ^a^ (Mann–Whitney test). Responders and non-responders indicate the subset of individuals in which αCGRP increased or not, respectively, at maximal intensity (see text).

**Table 2:** Metabolic and cardiorespiratory responses along the graded exercise test.

	BASELINE			VT1			VT2			MAXIMAL			RECOVERY ^a^		
	R (*n* = 8)	NR (*n* = 4)	*d* (CI95%)	R (*n* = 8)	NR (*n* = 4)	*d* (CI95%)	R (*n* = 8)	NR (*n* = 4)	*d* (CI95%)	R (*n* = 8)	NR (*n* = 4)	*d* (CI95%)	R (*n* = 8)	NR (*n* = 4)	*d* (CI95%)
**Time to (s)**	-	-	-	377.5 ± 63.7	177.5 ± 40.0	1.27 (0.09, 2.26)	940.0 ± 72.5	761.2 ± 117.0	0.83 (−0.27, 1.81)	1189.3 ± 52.0	992.5 ± 102.2	1.18 (0.02, 2.16)	405.0 ± 67.0	431.2 ± 113.4	−0.13 (−1.13, 0.89)
**Pulmonary function**															
Respiratory frequency (ventilations·min^−1^)	18.3 ± 1.5	16.5 ± 2.1	0.42 (−0.63, 1.40)	22.5 ± 1.1	20.7 ± 1.2	0.63 (−0.44, 1.61)	33.9 ± 2.7	28.8 ± 2.9	0.70 (−0.38, 1.68)	48.5 ± 1.7	42.1 ± 3.4	1.18 (0.02, 2.16)	22.20 ± 3.0	20.6 ± 3.0	−1.06 (−2.04, 0.08)
Tidal volume (L)	0.7 ± 0.1	0.6 ± 0.1	0.40 (−0.65, 1.38)	1.5± 0.1	1.4 ± 0.04	0.74 (−0.35, 1.71)	2.3 ± 0.1	2.5 ± 0.1	−0.57 (−1.55, 0.50)	2.5 ± 0.1	2.76 ± 0.1	−1.01 (−1.98, 0.12)	1.6 ± 0.1	1.6 ± 0.2	0.15 (−0.87, 1.14)
Minute ventilation (L·min^−1^)	12.9 ± 1.3	10.0 ± 0.8	0.88 (−0.23, 1.86)	34.9± 2.5	28.6 ± 1.7	1.02 (−0.12, 1.99)	78.5± 6.6	73.7 ± 9.6	0.25 (−0.78, 1.25)	123.9± 6.1	115.7 ± 8.3	0.48 (−0.58, 1.46)	35.4± 4.9	32.0 ± 6.2	0.26 (−0.77, 1.25)
**Cardiovascular function**															
Heart rate (beats·min^−1^)	73.3 ± 3.4	71.7 ± 8.1	0.13 (−0.88, 1.13)	117.7 ± 5.9	106.5 ± 5.3	1.47 (0.025, 2.46)	166.0 ± 2.4	160.0 ± 8.8	0.53 (−0.53, 1.51)	179.8 ± 1.8	177.7 ± 5.4	0.29 (−0.75, 1.27)	116.8 ± 3.9	114.2 ± 4.6	0.25 (−0.78, 1.24)
Stroke volume (mL)	96.2 ± 9.9	75.7 ± 6.8	0.83 (−0.27, 1.81)	133.2 ± 8.2	126.0 ± 7.1	0.35 (−0.69, 1.33)	126.4 ± 5.7	120.6 ± 4.9	0.40 (−0.65, 1.38)	125.5 ± 5.4	116.9 ± 4.1	0.63 (−0.45, 1.60)	87.1 ± 6.7	81.5 ± 3.9	0.34 (−0.70, 1.33)
Cardiac output (L·min^−1^)	6.9 ± 0.7	5.3 ± 0.6	0.85 (−0.26, 1.82)	15.4 ± 0.6	13.3 ± 0.5	1.28 (0.10, 2.26)	20.9 ± 0.9	19.3 ± 1.4	0.61 (−0.46, 1.59)	22.5 ± 0.9	20.8 ± 1.2	0.66 (−0.42, 1.64)	10.2 ± 0.8	9.3 ± 0.7	0.38 (−0.66, 1.37)
Systolic blood pressure (mmHg)	133.5 ± 6.2	139.2 ± 5.8	−0.33 (−10.34, 0.68)	164.8 ± 9.5 ^b^	150.6 ± 6.5 ^c^	0.72 (−0.44, 1.73)	174.1 ± 7.0 ^b^	141.0 ± 10.1 ^c^	1.91 (0.36, 3.03)	179.4 ± 5.6 ^d^	172.2 ± 14.9	0.33 (−0.73, 1.34)	140.6 ± 9.1	138.7 ± 4.6	0.08 (−0.93, 1.09)
Diastolic blood pressure (mmHg)	79.1 ± 3.2	78.7 ± 6.0	0.04 (−0.97, 1.04)	74.3 ± 7.9 ^b^	60.3 ± 6.1 ^c^	0.83 (−0.34, 1.85)	67.5 ± 5.1 ^b^	64.3 ± 8.9 ^c^	0.23 (−0.96, 1.37)	67.5 ± 4.1 ^d^	67.0 ± 4.1	−0.21 (−1.23, 0.84)	61.2 ± 4.5	51.5 ± 4.7	0.83 (−0.28, 1.80)
**Ventilatory exchange**															
VO_2_ (mL·min^−1^)	507.0 ± 65.3	374.7 ± 49.0	0.81 (−0.29, 1.78)	1641.5 ± 114.8	1311.3 ± 38.0	1.20 (0.03, 2.18)	3069.2 ± 171.7	2802.2 ± 273.7	0.53 (−0.53, 1.51)	3715.8 ± 156.9	3417.8 ± 199.3	0.69 (−0.39, 1.67)	844.2 ± 94.3	777.7 ± 107.2	0.26 (−0.77, 1.25)
VCO_2_ (mL·min^−1^)	409.3 ± 58.0	296.7 ± 31.2	0.80 (−0.30, 1.77)	1483.2 ± 127.0	1075.4 ± 48.8	1.34 (0.14, 2.32)	3226.6 ± 182.6	2902.2 ± 252.8	0.63 (−0.44, 1.61)	4234.6 ± 179.0	3829.9 ± 158.2	0.88 (−0.23, 1.86)	922.3 ± 137.3	822.5 ± 184.0	0.26 (−0.77, 1.25)
VO_2_ (mL·Kg^−1^·min^−1^)	6.5 ± 0.6	4.8 ± 0.6	0.99 (−0.14, 1.97)	21.9 ± 2.2	17.1 ± 0.9	0.98 (−0.15, 1.96)	40.3 ± 1.6	36.7 ± 4.0	0.61 (−0.46, 1.59)	48.8 ± 0.7	44.7 ± 3.2	1.02 (0−.11, 2.00)	10.9 ± 0.9	10.1 ± 1.3	0.30 (−0.74, 1.29)
VCO_2_ (mL·Kg^−1^·min^−1^)	5.21 ± 0.6	3.8 ± 0.3	0.97 (−0.16, 1.94)	19.7 ± 2.1	13.9 ± 0.9 *	1.11 (−0.04, 2.09)	42.1 ± 1.8	37.6 ± 3.5	0.78 (−0.32, 1.75)	55.3 ± 1.1	49.7 ± 2.7 *	1.45 (0.24, 2.44)	11.9 ± 1.5	10.6 ± 2.3	0.27 (−0.76, 1.26)
RER	0.8 ± 0.2	0.80 ± 0.04	0 (−1.01, 1.01)	0.89 ± 0.02	0.81 ± 0.01	1.71 (0.44, 2.72)	1.05 ± 0.01	1.04 ± 0.02	0.44 (−0.61, 1.42)	1.14 ± 0.01	1.12 ± 0.01	0.46 (−0.60, 1.44)	1.06 ± 0.06	1.02 ± 0.09	0.24 (−0.79, 1.23)
**Metabolism**															
Energy expenditure (Kcal·min^−1^)	2.4 ± 0.3	1.7 ± 0.2	0.81 (−0.29, 1.79)	8.1 ± 0.6	6.2 ± 0.2	1.24 (0.07, 2.22)	15.5 ± 0.8	14.2 ± 1.3	0.56 (−0.51, 1.54)	19.2 ± 0.8	17.6 ± 0.9	0.75 (−0.34, 1.73)	4.3 ± 0.5	3.9 ± 0.6	0.27 (−0.77, 1.26)
Energy from carbohydrates (%)	34.4 ± 6.9	36.6 ± 12.7	−0.10 (−1.10, 0.92)	65.9 ± 7.0	39.5 ± 5.7 *	1.47 (0.25, 2.47)	100	98.7 ± 1.2	0.91 (−0.20, 1.89)	100	100	-	87.0 ± 7.7	77.7 ± 12.9	0.40 (−0.64, 1.39)
**Work**															
Power (W)	-	-	-	112.5 ± 12.5	75.0 ± 10.2 *	1.19 (0.02, 2.17)	231.2 ± 16.8	193.7 ± 27.7 *	0.75 (−0.34, 1.72)	287.5 ± 11.5	243.7 ± 15.7 *	1.35 (0.16, 2.34)	-	-	-
METs	1.8 ± 0.1	1.4 ± 0.1	0.99 (−0.14, 1.97)	6.2 ± 0.5	4.9 ± 0.2	0.98 (−0.15, 1.96)	11.5 ± 0.4	10.4 ± 1.1	0.61 (−0.46, 1.59)	13.9 ± 0.2	12.7 ± 0.9	1.02 (−0.11, 2.00)	3.1 ± 0.2	2.9 ± 0.3	0.30 (−0.74, 1.29)

^a^ Time to recovery was measured from the end of the test. ^b^ *n* = 6. ^c^ *n* = 3. ^d^ *n* = 7. Between group differences at the corresponding condition are highlighted: * *p* < 0.05; 2-way RM ANOVA. R: Responders; NR: Non-responders; RER: respiratory exchange ratio; METs: metabolic equivalents.

## Data Availability

Data are available upon reasonable request to Moya-Ramón.

## Supplementary Materials

The following are available online at https://www.mdpi.com/article/10.3390/biology10080783/s1, Figure S1: Correlation between αCGRP and VO_2_, VCO_2_, carbohydrate oxidation (CHO) and relative power normalized to muscle mass.
