# Supplementary material for: Acute Increase in Blood αCGRP at Maximal Exercise and Its Association to Cardiorespiratory Fitness, Carbohydrate Oxidation and Work Performed: An Exploratory Study in Young Men
Source: Biology (Basel). 2021 Aug 17;10(8):783. doi: 10.3390/biology10080783 (PMC8389686; doi:10.3390/biology10080783)
Supplement: Supplementary file 1 [file biology-10-00783-s001.zip › biology-1316700-supplementary.pdf]

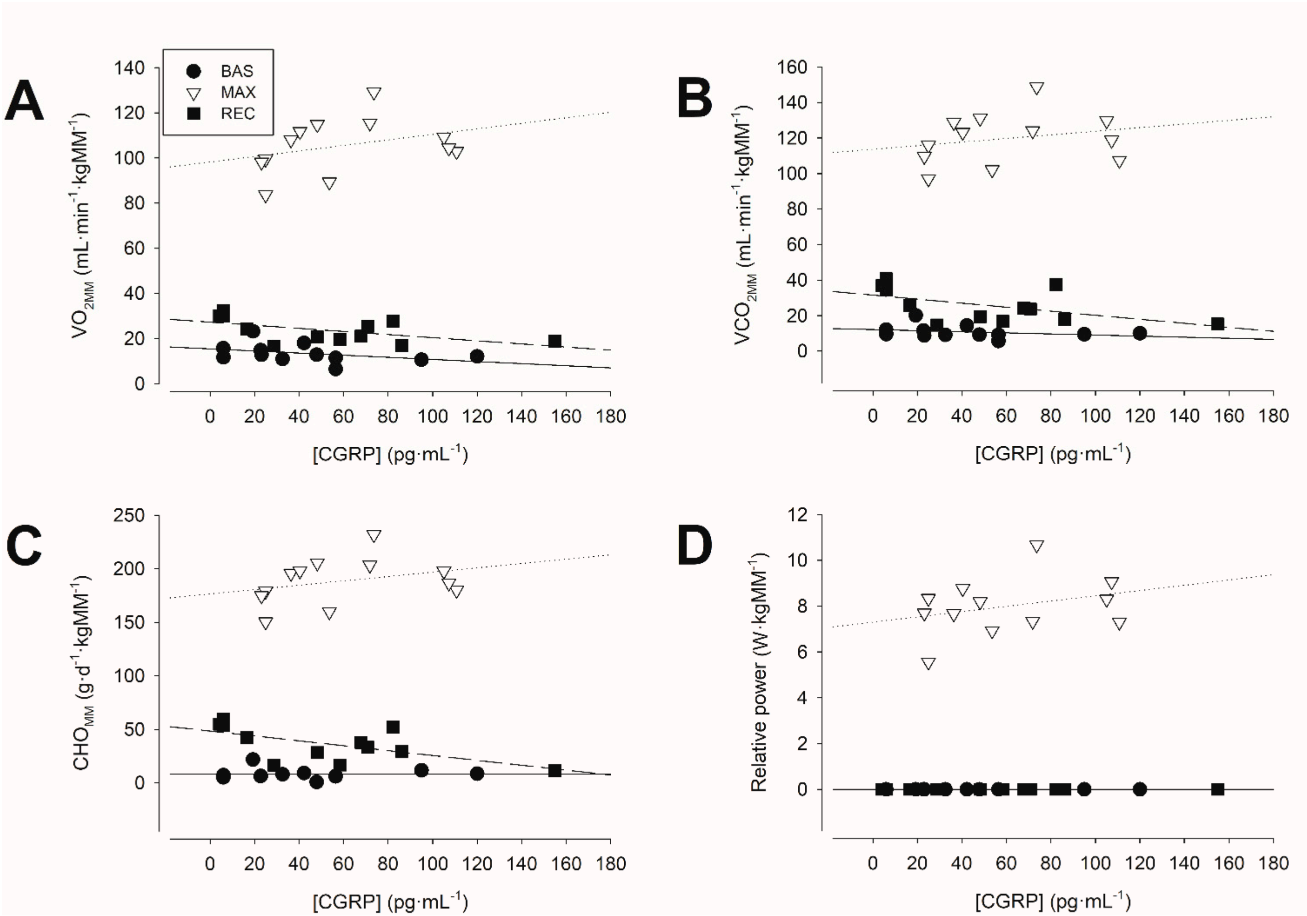

Figure S1. Correlation between αCGRP and VO_2, VCO_2, carbohydrate oxidation (CHO) and relative power normalized to muscle mass. Overall, αCGRP tended to correlate with these parameters only at maximal effort (MAX). **A.** Correlation of αCGRP and VO_{2MM} (at MAX: r=0.332, p=0.291); **B.** Correlation of αCGRP with VCO_{2MM} (at MAX: r=0.234, p=0.464); **C.** Correlation of αCGRP with carbohydrate oxidation (at MAX: r=0.308, p=0.329); **D.** Correlation of αCGRP with relative power (at MAX: r=0.304, p=0.336). Data corresponding to baseline are indicated by black circles and continuous lines; data corresponding to maximal effort, by open triangles and dotted lines, and data corresponding to the recovery by black squares and dashed lines. n = 12 for each condition in each panel; Pearson's correlation.
